# Changes in Influenza Vaccination Requirements for Health Care Personnel in US Hospitals

**DOI:** 10.1001/jamanetworkopen.2018.0143

**Published:** 2018-06-01

**Authors:** M. Todd Greene, Karen E. Fowler, David Ratz, Sarah L. Krein, Suzanne F. Bradley, Sanjay Saint

**Affiliations:** 1 Veterans Affairs Ann Arbor Healthcare System, Ann Arbor, Michigan; 2 Patient Safety Enhancement Program, Veterans Affairs Ann Arbor Healthcare System/University of Michigan, Ann Arbor; 3 Department of Internal Medicine, University of Michigan Medical School, Ann Arbor

## Abstract

**Importance:**

Annual influenza vaccinations are currently recommended for all health care personnel (HCP) to limit the spread of influenza to those at high risk of developing serious complications from the virus. Vaccination coverage has been shown to be significantly greater among employers requiring and encouraging HCP to receive the annual influenza vaccination.

**Objectives:**

To compare the proportion of respondent hospitals requiring HCP to receive annual influenza vaccination between 2013 and 2017 and to assess the degree to which these proportions differed between Veterans Affairs (VA) and non-VA hospitals.

**Design, Setting, and Participants:**

This national survey study included responses from 1062 infection preventionists between 2013 and 2017 from nationally representative samples of all VA and non-VA hospitals in the United States. Data analysis was conducted from November 17, 2017, to March 26, 2018.

**Main Outcomes and Measures:**

Survey response indicating hospital requirement for annual influenza vaccination of HCP.

**Results:**

The overall response rate for the 2013 survey was 69.3% (non-VA, 70.6% [403 of 571]; VA, 63.5% [80 of 126]) and in 2017 was 59.1% (non-VA, 59.1% [530 of 897]; VA, 58.9% [73 of 124]). Among all responding hospitals, mandatory influenza vaccination requirements for HCP increased from 37.1% in 2013 to 61.4% in 2017 (difference, 24.3%; 95% CI, 18.4%-30.2%; *P* < .001). This change was driven by non-VA hospitals, as requirement policies increased from 44.3% (171 of 386) in 2013 to 69.4% (365 of 526) in 2017 (difference, 25.1%; 95% CI, 18.8%-31.4%; *P* < .001). Conversely, there was no significant change during this period in the proportion of VA hospitals that required influenza vaccinations for HCP (1.3% [1 of 77] to 4.1% [3 of 73]; difference, 2.8%; 95% CI, −2.4% to 8.0%; *P* = .29).

**Conclusions and Relevance:**

Despite a substantial increase in mandates among non-VA hospitals, we found that many non-VA hospitals and nearly all VA hospitals are still not currently mandating influenza vaccinations for HCP. In addition to implementing other well-described strategies to increase vaccination rates, health care organizations should consider mandating influenza vaccinations while appropriately weighing and managing the moral, ethical, and legal implications.

## Key Points

**Question:**

How has the proportion of US hospitals requiring receipt of annual influenza vaccination among health care personnel changed in recent years?

**Findings:**

In this national survey study, which included responses from 1062 infection preventionists at both Veterans Affairs and non–Veterans Affairs hospitals between the 2013 and 2017 calendar years, required influenza vaccinations among health care personnel increased from 37.1% to 61.4%; this change was driven by increases in non–Veterans Affairs hospitals.

**Meaning:**

Influenza vaccination mandates for health care personnel have increased in recent years, coinciding with concurrent increases in vaccination coverage among health care personnel.

## Teaser

This national survey study investigates changes in the proportion of hospitals requiring health care personnel to receive annual influenza vaccinations in 2013 and 2017 and compares the change among Veterans Affairs and non–Veterans Affairs hospitals.

## Introduction

Annually, influenza accounts for significant morbidity, mortality, and cost burden.^1^ Since the 2010 to 2011 influenza season, estimates indicate that seasonal influenza-related illnesses ranged from 9.2 million to 35.6 million, with up to 710 000 hospitalizations leading to as many as 16.7 million medical visits and up to 20 000 pneumonia- and influenza-related deaths.^2^ Similar clinical and economic burdens were reported within the Veterans Affairs (VA) hospital setting.^3,4^ During the 2014 to 2015 influenza season, more than 11 500 confirmed cases and a hospitalization rate of 74.2 per 100 000 VA users were observed.^4^ Recent estimates of health care–associated influenza range between 1% and 5%.^5,6^ The Centers for Disease Control and Prevention Advisory Committee on Immunization Practices continues to recommend that all health care personnel (HCP) be vaccinated annually against influenza.^7^ Additionally, the US Department of Health and Human Services wants 90% of HCP vaccinated by 2020.^8^ During the 2016 to 2017 influenza season, an estimated 78.6% of all HCP received the annual influenza vaccination, with the highest coverage rates found among HCP required by their employer to be vaccinated (96.7%).^9^

Although multiple national recommendations urge influenza vaccination for all HCP,^10^ not all health care facilities mandate its receipt. As part of the national surveys of infection preventionists (experts on practical methods of preventing and controlling the spread of infectious diseases) conducted in 2013 and 2017,^11,12^ we assessed the degree to which hospitals require HCP to receive annual influenza vaccination. As we were interested in determining how influenza vaccination requirements for HCP have changed in recent years, we compared the proportion of respondent hospitals requiring HCP to receive annual influenza vaccination, based on survey responses from 2013 and 2017. We also assessed the degree to which these proportions differed between VA and non-VA hospitals.

## Methods

### Survey Procedures

This study was part of an ongoing panel survey in which we ask infection preventionists across the United States every 4 years what practices their hospitals are using to prevent common health care–associated infections.^11^ The study follows the American Association for Public Opinion Research (AAPOR) reporting guideline. For the first wave in 2005, the national random sample was selected by identifying all nonfederal, general medical, and surgical hospitals with an intensive care unit and at least 50 hospital beds using the 2003 American Hospital Association (AHA) database. Hospitals were then stratified in to 2 bed size groups (50-250 beds and ≥251 beds), and a random sample of 300 hospitals from each group was selected. For the second (2009) and third (2013) waves of the ongoing study, the survey was sent to the same hospitals sampled in 2005 with a few exceptions owing to closures or mergers between the longitudinal survey points. Because data from the 2003 AHA database may no longer reflect the current distribution of US hospitals, for the fourth (2017) wave we resampled based on AHA fiscal year 2013 data. In the fourth wave, we randomly sampled 900 general medical and surgical hospitals with an intensive care unit. Hospitals of all bed sizes were included in the 2017 sample. For each survey wave, we sent surveys to all VA hospitals across the United States. This study obtained institutional review board exemption from the University of Michigan and approval from the VA Ann Arbor Healthcare System. The VA survey was anonymous and was conducted with a waiver of signed informed consent.

The study surveys were mailed to the hospital infection preventionist. At hospitals that employ more than 1 infection preventionist, we asked that the lead infection preventionist serve as the primary respondent, although we encouraged consulting with others as needed to complete the questionnaire. The survey process followed a modified Dillman approach,^13^ which included a first mailing of the survey, a reminder letter or postcard after approximately 2 weeks, and additional survey mailings at 4, 7, and 16 weeks to infection preventionists who had not yet responded.

The survey instrument included questions about facility characteristics, the infection control program, infection preventionists, and frequency of use of practices related to the prevention of health care–associated infections. The third wave, distributed in May 2013, also included the question, “Are health care workers at your hospital who provide patient care required to receive influenza vaccination?” Respondents answering no to this question were asked to specify the reason HCP were not required to receive the influenza vaccination. The fourth wave, distributed in May 2017, included the question, “Does your hospital mandate health care workers to receive annual influenza vaccination?” along with questions regarding declination and requirements for wearing masks.

### Statistical Analysis

As only the third and fourth waves of the survey included questions regarding influenza vaccination requirements for HCP, the current analysis compares responses from these waves. Descriptive statistics were generated for select general hospital characteristics obtained from the 2013 AHA survey and for the respective proportions of hospitals requiring annual influenza vaccination for HCP. Confidence intervals were calculated using Proc Freq (SAS Institute Inc) with the RISKDIFF statement, which gives the 95% Wald confidence interval based on asymptotic standard errors. To determine differences in proportions, we used the χ^2^ test. All tests were 2-sided with a *P* value less than .05 considered statistically significant. All analyses were conducted using SAS software version 9.4 (SAS Institute Inc).

## Results

The overall response rate for the 2013 survey was 69.3% (non-VA, 70.6% [403 of 571]; VA, 63.5% [80 of 126]) and in 2017 was 59.1% (non-VA, 59.1% [530 of 897]; VA, 58.9% [73 of 124]). A comparison of respondent and nonrespondent characteristics for both waves of the survey is provided in the eTable in the Supplement.

A total of 1062 hospitals (463 in 2013 and 599 in 2017) were included in this analysis; 24 hospitals (20 from 2013 and 4 from 2017) did not answer the influenza vaccination questions and were removed. Select hospital characteristics by VA status and survey year are shown in Table 1. Approximately 60% of VA hospitals that participated in this study were in rural locations (2013: 19 of 31 [61%]; 2017: 18 of 30 [60%]) and 80% were teaching hospitals (2013: 65 of 79 [82.3%]; 2017: 56 of 72 [77.8%]). There were some differences seen in the participating non-VA hospital demographic characteristics between 2013 and 2017. In 2017, there were fewer urban hospitals (2013: 300 of 358 [83.8%]; 2017: 413 of 528 [78.2%]; difference, −5.6%; 95% CI, −10.8% to −0.4%; *P* = .04) and teaching hospitals (2013: 161 of 400 [40.3%]; 2017: 170 of 528 [32.2%]; difference, −8.1%; 95% CI, −14.3% to −1.8%; *P* = .01). In 2017, non-VA hospitals also had a lower average number of hospital beds (mean [SD], 2013: 273.0 [214.3] beds; 2017: 202.6 [189.5] beds; difference, −70.3%; 95% CI, −96.8 to −43.8%; *P* < .001). Overall, the percentage of hospitals reporting mandatory influenza vaccinations for HCP increased from 37.1% in 2013 to 61.4% in 2017 (difference, 24.3%; 95% CI, 18.4%-30.2%; P < .001) (non-VA: 44.3% [171 of 386] in 2013 to 69.4% [365 of 526] in 2017; difference, 25.1%; 95% CI, 18.8%-31.4%; P < .001; VA: 1.3% [1 of 77] in 2013 to 4.1% [3 of 73] in 2017; difference, 2.8%; 95% CI, −2.4% to 8.0%; *P* = .29) (Figure).

**Table 1.** Hospital Characteristics by VA Status and Survey Year

Characteristic	Hospital Type, No./Total No. (%)					
	Non-VA			VA		
	2013	2017	*P* Value	2013	2017	*P* Value
Location						
Urban	300/358 (83.8)	413/528 (78.2)	.04	12/31 (38.7)	12/30 (40.0)	.92
Rural	58/358 (16.2)	115/528 (21.8)		19/31 (61.3)	18/30 (60.0)	
Profit status						
For profit	45/400 (11.3)	60/528 (11.4)	.96	0	0	NA
Nonprofit	355/400 (88.8)	468/528 (88.6)		80/80 (100)	73/73 (100)	
Teaching						
Yes	161/400 (40.3)	170/528 (32.2)	.01	65/79 (82.3)	56/72 (77.8)	.49
No	239/400 (59.8)	358/528 (67.8)		14/79 (17.7)	16/72 (22.2)	
Hospital beds, mean (SD), No.	273.0 (214.3)	202.6 (189.5)	<.001	240.3 (195.9)	229.3 (177.2)	.72
Vaccination mandates	171 (44.3)	365 (69.4)	<.001	1 (1.3)	3 (4.1)	.29

Abbreviations: NA, not applicable; VA, Veterans Affairs.

**Figure.**
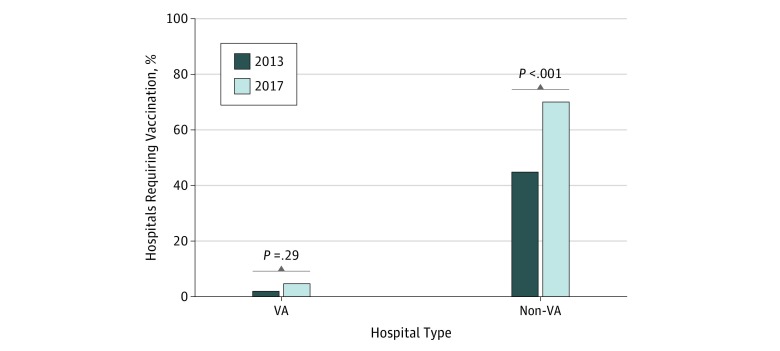
Changes in Health Care Personnel Influenza Vaccination Mandate by VA Status VA indicates Veterans Affairs.

Table 2 shows differences in mandate presence by survey year and hospital characteristics among non-VA hospitals. Because too few VA hospitals mandated vaccinations, we were unable to stratify results among VA hospitals based on hospital characteristics. Differences in the reported presence of mandates by hospital characteristics were not observed among 2013 respondents from non-VA hospitals. In 2017, mandates for HCP influenza vaccinations were reported by a higher percentage of nonprofit hospitals compared with their for-profit counterparts (difference, 25.5%; 95% CI, 12.3%-38.8%; *P* < .001).

**Table 2.** Non–Veterans Affairs Hospitals Health Care Personnel Influenza Vaccination Mandate by Survey Year and Hospital Characteristic

Characteristic	No./Total No. (%) by Survey Year					
	2013			2017		
	Mandate	No Mandate	*P* Value	Mandate	No Mandate	*P* Value
Location						
Urban	135/157 (86.0)	151/186 (81.2)	.23	286/363 (78.8)	124/161 (77.0)	.65
Rural	22/157 (14.0)	35/186 (18.8)		77/363 (21.2)	37/161 (23.0)	
Profit status						
For profit	16/170 (9.4)	29/213 (13.6)	.20	28/363 (7.7)	32/161 (19.9)	<.001
Nonprofit	154/170 (90.6)	184/213 (86.4)		335/363 (92.3)	129/161 (80.1)	
Teaching						
Yes	74/170 (43.5)	79/213 (37.1)	.20	115/363 (31.7)	53/161 (32.9)	.78
No	96/170 (56.5)	134/213 (62.9)		248/363 (68.3)	108/161 (67.1)	
Hospital beds, mean (SD), No.	292.8 (216.6)	255.9 (210.1)	.09	204.5 (189.2)	197.8 (186.6	.71

Aspects of hospital influenza vaccination policies by mandate status for 2017 are shown in Table 3. All 368 hospitals mandating influenza vaccinations had allowable declinations. Of note, 94 of 366 hospitals (25.7%) with vaccination mandates in place did not impose penalties for noncompliance with the hospital policy on influenza vaccination. Among the 231 hospitals that reported not having a specific influenza vaccination mandate, 158 hospitals (68.4%) had policies for declination (non-VA: 75.2% [121 of 161]; VA: 52.9% [37 of 70]) and 94 hospitals (40.9%) had requirements for wearing masks (non-VA: 57.8% [93 of 161]; VA: 1.4% [1 of 69]).

**Table 3.** Aspects of Hospital Health Care Personnel Annual Influenza Vaccination Policies in 2017

Policy	No./Total No. (%)		
	Vaccination Mandate (n = 368)	No Vaccination Mandate (n = 231)	Total (n = 599)
Allowable reasons to decline influenza vaccination^a^	368/368 (100)	158/231 (68.4)	526/599 (87.8)
Medical contraindication	354/368 (96.2)	130/231 (56.3)	484/599 (80.8)
Religious reasons	287/368 (78.0)	102/231 (44.2)	389/599 (64.9)
No reason required	47/368 (12.8)	75/231 (32.5)	122/599 (20.4)
Other	29/368 (7.9)	22/231 (9.5)	51/599 (8.5)
Require mask when providing patient care during influenza season if not vaccinated	305/368 (82.9)	94/230 (40.9)	399/598 (66.7)
Penalties for noncompliance with hospital policy on influenza vaccination	272/366 (74.3)	49/228 (21.5)	321/594 (54.0)

^a^ Respondents could select more than 1 answer.

## Discussion

Several important findings emerged from our national survey study. First, compared with 2013, when less than half of hospitals mandated influenza vaccinations,^12^ there was a significant increase in hospitals mandating HCP receive vaccinations in 2017 among non-VA hospitals. Second, despite an increase in the proportion of non-VA hospitals mandating vaccinations, penalties for noncompliance with the hospital policy on influenza vaccination were not universal among hospitals with mandates. Third, few VA hospitals mandated receipt of HCP influenza vaccination, and we did not observe a significant change between 2013 and 2017. While the VA did not specifically mandate receiving influenza vaccinations at the time of the 2013 and 2017 surveys, a VA directive was subsequently released in September 2017^14^ articulating that all HCP were expected to receive the annual influenza vaccination and were required to wear masks throughout the influenza season if unable or unwilling to get vaccinated.

Mandating influenza vaccination remains a controversial topic, with uncertainty of the effectiveness of HCP influenza vaccination in reducing patient morbidity and mortality,^6,15,16,17,18,19,20^ different conclusions regarding the grading of the evidence,^21,22^ and numerous legal and ethical precedents to be carefully considered.^23,24,25,26^ Still, over the past several years, HCP influenza vaccination coverage rates have continuously been greater than 95% among HCP required by their employer to be vaccinated.^9,27,28^

Although mandating influenza vaccinations intuitively leads to increased vaccination coverage of HCP, other strategies have proven to be successful. Strategies to promote influenza vaccinations, including influenza education for HCP, free and easily accessible vaccinations, annual influenza campaigns, incentives, signed declination policies, and using HCP vaccination rates as an organizational quality measure, have been well described.^29,30,31,32^ Starting January 2013, acute care hospitals participating in the Centers for Medicare & Medicaid Hospital Inpatient Quality Reporting Program were required to report HCP influenza vaccination data through the Centers for Disease Control and Prevention National Healthcare Safety Network program.^33^ Public reporting of vaccination data has been shown to increase vaccination coverage,^34^ and it is plausible that the increase in vaccination mandates observed in this study stems, at least in part, from the contemporaneous implementation of the Centers for Medicare & Medicaid public reporting ruling.

We found that few VA hospitals explicitly mandated that HCP receive annual influenza vaccination. As outlined in the goals of the 2017 to 2018 VA influenza vaccination program,^35^ since the beginning of fiscal year 2013 VA facilities have been expected to gradually work toward the 2020 Healthy People goal of 90% influenza vaccination rate for HCP. Several VA hospitals have previously shown success in improving HCP vaccination rates in the absence of a national mandate. For example, the Minneapolis VA Health Care System in Minneapolis, Minnesota, improved vaccination rates from less than 25% to greater than 65% by implementing the use of mobile carts to facilitate the delivery of vaccinations.^36^ The Lebanon VA Medical Center in Lebanon, Pennsylvania, increased vaccination rates from approximately 50% to greater than 75% by offering time-off incentives to vaccine recipients.^29^ Regardless of whether an organization has an official mandate for vaccinations, establishing a written policy that states the organizational commitment to increasing vaccination rates is among the recommended strategies for improving vaccination coverage among HCP.^29^

### Limitations

Our study had limitations. First, the survey response rates were 69% in 2013 and 59% in 2017, and our results may not be generalizable to all hospitals. Second, the updated 2017 responses were obtained from a different sample of hospitals than in previous waves, with a statistically significantly higher proportion of rural, nonteaching hospitals with smaller total bed sizes. Still, this updated sample is nationally representative, and we did not detect differences in the presence of vaccination mandates by these hospital characteristics. Third, it is possible that the observed increase in the proportion of non-VA hospitals mandating vaccinations between surveys could have partially stemmed from the slight difference in question wording. Of note, the 2013 survey question explicitly focused on HCP providing patient care, potentially leading to an underestimation of the vaccination requirement among all HCP. This may have been represented more in 2017 responses to the reworded question, which lacked explicit mention of patient care provision. In the 2017 survey, although the provision of patient care was not specified directly in our key question of interest, it was included in the question regarding the requirement for wearing a mask for HCP not receiving the influenza vaccination. As such, these details may have helped prompt the respondent to think of HCP providing direct patient care. Fourth, it is possible that our study of the proportion of hospitals having a mandate in place for HCP vaccination is underreported, as the presence of declination policies or requirement for wearing a mask could be considered mandates. However, this potential underreporting likely applied equivalently in both 2013 and 2017 survey years. Fifth, we did not collect influenza infection rate data for the surveyed hospitals. As such, we were not able to demonstrate whether influenza rates differed by mandatory vaccination status.

## Conclusions

This 2017 US national survey found that more than two-thirds of non-VA hospitals mandate HCP influenza vaccination, which is a significant increase from 4 years prior. While HCP influenza vaccination in VA hospitals is strongly encouraged, as of summer 2017, less than 5% of VA hospitals mandated influenza vaccination for HCP providing care for veterans.

## Notes

Supplement. **eTable.** Comparison of Survey Respondents and Non-Respondents by Veterans Affairs Status and Survey Year

## References

[1] Molinari NA, Ortega-Sanchez IR, Messonnier ML, The annual impact of seasonal influenza in the US: measuring disease burden and costs. Vaccine. 2007;25(27):-. 17544181 10.1016/j.vaccine.2007.03.046

[2] Rolfes MA, Foppa IM, Garg S, Annual estimates of the burden of seasonal influenza in the United States: a tool for strengthening influenza surveillance and preparedness. Influenza Other Respir Viruses. 2018;12(1):132-137. 29446233 10.1111/irv.12486 PMC5818346

[3] Young-Xu Y, van Aalst R, Russo E, Lee JK, Chit A The annual burden of seasonal influenza in the US Veterans Affairs population. PLoS One. 2017;12(1):e0169344. 28046080 10.1371/journal.pone.0169344 PMC5207669

[4] Lucero-Obusan C, Schirmer PL, Wendelboe A, Oda G, Holodniy M Epidemiology and burden of influenza in the US Department of Veterans Affairs. Influenza Other Respir Viruses. 2018;12(2):293-298. 29045064 10.1111/irv.12512 PMC5820422

[5] Cummings CN, Garg S, Nenninger EK, Hospital-acquired influenza among hospitalized patients, 2011-2015. Open Forum Infect Dis. 2016;3(suppl 1):1742. doi:10.1093/ofid/ofw194.122

[6] Dionne B, Brett M, Culbreath K, Mercier RC Potential ceiling effect of healthcare worker influenza vaccination on the incidence of nosocomial influenza infection. Infect Control Hosp Epidemiol. 2016;37(7):840-844. 27098758 10.1017/ice.2016.77

[7] Grohskopf LA, Sokolow LZ, Broder KR, Prevention and control of seasonal influenza with vaccines: recommendations of the advisory committee on immunization practices—United States, 2017-18 influenza season. MMWR Recomm Rep. 2017;66(2):1-20. 28841201 10.15585/mmwr.rr6602a1 PMC5837399

[8] Office of Disease Prevention and Health Promotion Healthy People 2020 topics and objectives: immunization and infectious diseases: topic IID-12.9. https://www.healthypeople.gov/2020/topics-objectives/topic/immunization-and-infectious-diseases/objectives. Accessed March 26, 2018.

[9] Black CL, Yue X, Ball SW, Influenza vaccination coverage among health care personnel—United States, 2016-17 influenza season. MMWR Morb Mortal Wkly Rep. 2017;66(38):1009-1015. 28957042 10.15585/mmwr.mm6638a1 PMC5657674

[10] Infectious Diseases Society of America; Society for Healthcare Epidemiology of America; Pediatric Infectious Diseases Society IDSA, SHEA, and PIDS joint policy statement on mandatory immunization of health care personnel according to the ACIP-recommended vaccine schedule. http://www.idsociety.org/uploadedFiles/IDSA/Policy_and_Advocacy/Current_Topics_and_Issues/Immunizations_and_Vaccines/Health_Care_Worker_Immunization/Statements/IDSA_SHEA_PIDS%20Policy%20on%20Mandatory%20Immunization%20of%20HCP.pdf. Published December 2013. Accessed March 26, 2018.

[11] Krein SL, Fowler KE, Ratz D, Meddings J, Saint S Preventing device-associated infections in US hospitals: national surveys from 2005 to 2013. BMJ Qual Saf. 2015;24(6):385-392. 25862757 10.1136/bmjqs-2014-003870

[12] Greene MT, Fowler KE, Krein SL, Influenza vaccination requirements for healthcare personnel in US hospitals: results of a national survey. Infect Control Hosp Epidemiol. 2016;37(4):485-487. 26996061 10.1017/ice.2015.277

[13] Dillman DA Mail and Internet Surveys: The Tailored Design Method. 2nd ed New York, NY: John Wiley & Sons; 2000.

[14] US Department of Veterans Affairs VHA directive 1192: seasonal influenza prevention program for VHA health care personnel. https://www.publichealth.va.gov/docs/flu/VHA_Directive_1192_Sep2017.pdf. Published September 26, 2017. Accessed March 26, 2018.

[15] De Serres G, Skowronski DM, Ward BJ, Influenza vaccination of healthcare workers: critical analysis of the evidence for patient benefit underpinning policies of enforcement. PLoS One. 2017;12(1):e0163586. 28129360 10.1371/journal.pone.0163586 PMC5271324

[16] Amodio E, Restivo V, Firenze A, Mammina C, Tramuto F, Vitale F Can influenza vaccination coverage among healthcare workers influence the risk of nosocomial influenza-like illness in hospitalized patients? J Hosp Infect. 2014;86(3):182-187. 24581755 10.1016/j.jhin.2014.01.005

[17] Carman WF, Elder AG, Wallace LA, Effects of influenza vaccination of health-care workers on mortality of elderly people in long-term care: a randomised controlled trial. Lancet. 2000;355(9198):93-97. 10675165 10.1016/S0140-6736(99)05190-9

[18] Hayward AC, Harling R, Wetten S, Effectiveness of an influenza vaccine programme for care home staff to prevent death, morbidity, and health service use among residents: cluster randomised controlled trial. BMJ. 2006;333(7581):1241. 17142257 10.1136/bmj.39010.581354.55 PMC1702427

[19] Lemaitre M, Meret T, Rothan-Tondeur M, Effect of influenza vaccination of nursing home staff on mortality of residents: a cluster-randomized trial. J Am Geriatr Soc. 2009;57(9):1580-1586. 19682118 10.1111/j.1532-5415.2009.02402.x

[20] Potter J, Stott DJ, Roberts MA, Influenza vaccination of health care workers in long-term-care hospitals reduces the mortality of elderly patients. J Infect Dis. 1997;175(1):1-6. 8985189 10.1093/infdis/175.1.1 PMC7109672

[21] Ahmed F, Lindley MC, Allred N, Weinbaum CM, Grohskopf L Effect of influenza vaccination of healthcare personnel on morbidity and mortality among patients: systematic review and grading of evidence. Clin Infect Dis. 2014;58(1):50-57. 24046301 10.1093/cid/cit580

[22] Thomas RE, Jefferson T, Lasserson TJ Influenza vaccination for healthcare workers who care for people aged 60 or older living in long-term care institutions. Cochrane Database Syst Rev. 2013;(7):CD005187. 23881655 10.1002/14651858.CD005187.pub4

[23] National Center for Ethics in Health Care Strategies to increase influenza vaccination rates among health care workers: ethical considerations. National Ethics Teleconference. http://www.ethics.va.gov/docs/net/NET_Topic_20080130_Strategies_in_increasing_influenza_vaccination_rates_in_health_care_workers-Ethical_Considerations.doc. Published January 30, 2008. Accessed March 26, 2018.

[24] Wang TL, Jing L, Bocchini JA Jr Mandatory influenza vaccination for all healthcare personnel: a review on justification, implementation and effectiveness. Curr Opin Pediatr. 2017;29(5):606-615. 28700416 10.1097/MOP.0000000000000527

[25] Talbot TR Update on immunizations for healthcare personnel in the United States. Vaccine. 2014;32(38):4869-4875. 24231439 10.1016/j.vaccine.2013.10.090

[26] Dubov A, Phung C Nudges or mandates? the ethics of mandatory flu vaccination. Vaccine. 2015;33(22):2530-2535. 25869886 10.1016/j.vaccine.2015.03.048

[27] Black CL, Yue X, Ball SW, ; Centers for Disease Control and Prevention (CDC) Influenza vaccination coverage among health care personnel—United States, 2013-14 influenza season. MMWR Morb Mortal Wkly Rep. 2014;63(37):805-811. 25233281 PMC5779456

[28] Black CL, Yue X, Ball SW, Influenza vaccination coverage among health care personnel—United States, 2014-15 influenza season. MMWR Morb Mortal Wkly Rep. 2015;64(36):993-999. 26389743 10.15585/mmwr.mm6436a1

[29] The Joint Commission Providing a Safer Environment for Health Care Personnel and Patients Through Influenza Vaccination: Strategies From Research and Practice. Oakbrook Terrace, IL: The Joint Commission; 2009.

[30] Polgreen PM, Chen Y, Beekmann S, ; Infectious Diseases Society of America’s Emerging Infections Network Elements of influenza vaccination programs that predict higher vaccination rates: results of an emerging infections network survey. Clin Infect Dis. 2008;46(1):14-19. 18171207 10.1086/523586

[31] Simeonsson K, Summers-Bean C, Connolly A Influenza vaccination of healthcare workers: institutional strategies for improving rates. N C Med J. 2004;65(6):323-329. 15714719

[32] Talbot TR, Dellit TH, Hebden J, Sama D, Cuny J Factors associated with increased healthcare worker influenza vaccination rates: results from a national survey of university hospitals and medical centers. Infect Control Hosp Epidemiol. 2010;31(5):456-462. 20233060 10.1086/651666

[33] Centers for Medicare & Medicaid Services Medicare program; hospital inpatient prospective payment systems for acute care hospitals and the long-term care hospital prospective payment system and FY 2012 rates; hospitals’ FTE resident caps for graduate medical education payment, 42 CFR parts 412, 413, and 476. Vol 76 Washington, DC: Department of Health and Human Services; 2011 https://www.cms.gov/Medicare/Medicare-Fee-for-Service-Payment/AcuteInpatientPPS/FY-2012-IPPS-Final-Rule-Home-Page-Items/CMS1250103.html. 21894648

[34] Lindley MC, Bridges CB, Strikas RA, ; Centers for Disease Control and Prevention Influenza vaccination performance measurement among acute care hospital-based health care personnel—United States, 2013-14 influenza season. MMWR Morb Mortal Wkly Rep. 2014;63(37):812-815. 25233282 PMC5779458

[35] US Department of Veterans Affairs Goals for 2017-2018 VA influenza vaccination program. https://www.publichealth.va.gov/flu/professionals/goals.asp. Accessed March 26, 2018.

[36] Centers for Disease Control and Prevention Interventions to increase influenza vaccination of health care workers—California and Minnesota. MMWR Morb Mortal Wkly Rep. 2005;54(8):196-199. 15744227

