# Supplementary material for: Changes in Influenza Vaccination Requirements for Health Care Personnel in US Hospitals
Source: JAMA Netw Open. 2018 Jun 1;1(2):e180143. doi: 10.1001/jamanetworkopen.2018.0143 (PMC6324418; doi:10.1001/jamanetworkopen.2018.0143)
Supplement: Supplement. — eTable. Comparison of Survey Respondents and Non-Respondents by Veterans Affairs Status and Survey Year [file jamanetwopen-1-e180143-s001.pdf]

Supplementary Online Content

Greene MT, Fowler KE, Ratz D, Krein SL, Bradley SF, Saint S. Changes in influenza vaccination requirements for health care personnel in US hospitals. *JAMA Netw Open*. 2018;1(2):e180143. doi:10.1001/jamanetworkopen.2018.0143

eTable. Comparison of Survey Respondents and Non-Respondents by Veterans Affairs Status and Survey Year

This supplementary material has been provided by the authors to give readers additional information about their work.

© 2018 Greene MT et al. *JAMA Network Open*.

eTable. Comparison of Survey Respondents and Non-Respondents by Veterans Affairs Status and Survey Year

Non-VA	2013			2017		
Characteristic	Respondent	Non-respondent	P	Respondent	Non-respondent	P
Location						
Urban	300 (83.8%)	122 (84.7%)	0.8	413 (78.2%)	312 (84.8%)	0.01
Rural	58 (16.2%)	22 (15.3%)		115 (21.8%)	56 (15.2%)	
Profit Status						
For profit	45 (11.3%)	23 (14.1%)	0.34	60 (11.4%)	48 (13.0%)	0.46
Non-profit	355 (88.8%)	140 (85.9%)		468 (88.6%)	321 (87.0%)	
Teaching						
Yes	161 (40.3%)	80 (49.1%)	0.05	170 (32.2%)	137 (37.1%)	0.13
No	239 (59.8%)	83 (50.9%)		358 (67.8%)	232 (62.9%)	
Hospital Beds	273.0 ± 214.3	332.4 ± 295.2	0.02	202.6 ± 189.5	237.8 ± 215.0	0.01
VA	2013			2017		
Characteristic	Respondent	Non-respondent	P	Respondent	Non-respondent	P
Location						
Urban	12 (38.7%)	9 (45.0%)	0.66	12 (40.0%)	8 (40.0%)	0.99
Rural	19 (61.3%)	11 (55.0%)		18 (60.0%)	12 (60.0%)	
Teaching						
Yes	65 (82.3%)	38 (84.4%)	0.76	56 (77.8%)	44 (88.0%)	0.15
No	14 (17.7%)	7 (15.6%)		16 (22.2%)	6 (12.0%)	
Hospital beds	240.3 ± 195.9	290.4 ± 266.3	0.27	229.3 ± 177.2	305.6 ± 276.6	0.09

© 2018 Greene MT et al. *JAMA Network Open.*
